# Antibiotic Resistance in Salmonella typhi Strains Isolated From Patients in Pakistan: A Hospital Database Study

**DOI:** 10.7759/cureus.58240

**Published:** 2024-04-14

**Authors:** Muhammad Yousaf, Irshad Sikandar, Zeeshan Waqas, Sara Pervez, Hussam Jehanzeb, Ameer M Farrukh, Yaxel Levin-Carrion, Bader Semakieh, Qaisar Ali Khan

**Affiliations:** 1 Medicine, Medical Teaching Institute, Khyber Teaching Hospital (MTI/KTH), Peshawar, PAK; 2 Cardiology, Peshawar Institute of Cardiology, Peshawar, PAK; 3 Dermatology, Medical Teaching Institute, Khyber Teaching Hospital (MTI/KTH), Peshawar, PAK; 4 Medicine, Hayatabad Medical Complex Peshawar, Peshawar, PAK; 5 Medicine, University of Galway, Galway, IRL; 6 Medicine, Rutgers University New Jersey Medical School, Newark, USA; 7 Neurology, Arkansas College of Osteopathic Medicine, Arkansas, USA; 8 Internal Medicine, Medical Teaching Institute, Khyber Teaching Hospital (MTI/KTH), Peshawar, PAK; 9 Internal Medicine, District Headquarter (DHQ) Teaching Hospital KDA Kohat, Kohat, PAK

**Keywords:** enteric fever (typhoid fever), pakistan, multi-drug resistance, antibiotic resistance, salmonella typhi

## Abstract

Background: The surge in antibiotic-resistant *Salmonella enterica* serotype Typhi strains has led to heightened morbidity, mortality, and treatment expenses. This study aims to assess the resistance patterns of *Salmonella* Typhi to diverse antibiotics among patients seeking care at a tertiary hospital in Pakistan.

Methods: A database from a tertiary care hospital in Pakistan was reviewed, and data on blood cultures that isolated *Salmonella enterica* serotype Typhi were collected. Data were collected and analyzed using Microsoft Excel (Microsoft Corporation, USA) and IBM SPSS software (IBM Corp., Armonk, NY).

Results: Demographic information of the selected data was retrieved from the hospital database, and the results showed that 63.7% were male, 36.1% were female, and 0.2% were categorized as neutered. Regarding antibiotic resistance, ampicillin exhibited the highest resistance rate (91.50%), while meropenem demonstrated the lowest (3.00%). Antibiotic sensitivity patterns also varied across different age groups, although statistical analysis indicated no significant differences. Significant associations were found between antibiotic resistance and comorbidities, as well as previous antibiotic use.

Conclusion: *Salmonella enterica* serotype Typhi showed a high resistance to ampicillin and fluoroquinolones, such as ciprofloxacin. The emergence of resistance and decreased sensitivity to current first-line antibiotics necessitates a shift towards alternative options, such as third-generation cephalosporins, azithromycin, and newer antibiotics like meropenem.

## Introduction

Typhoid fever caused by the bacterium *Salmonella* Typhi has been a significant public health problem in many parts of the world for centuries. The annual incidence of typhoid fever around the world is approximately 12-27 million cases [1]. It is a food-borne pathogen that is responsible for a wide range of illnesses, ranging from gastroenteritis to sepsis [2]. In 2000, enteric fever caused 200,000 deaths worldwide, predominantly in underdeveloped countries [3]. The incidence of enteric fever is comparatively lower in the USA and some European countries. Many Asian countries have high incidence rates of enteric fever, especially Pakistan and India. However, the true incidence of enteric fever is often underestimated because of limited diagnostic resources and proper surveillance tools in endemic regions. Several epidemiological studies for the past few years show that in China and Vietnam, the number of children with enteric fever annually was 25 per 100,000, while in India and Pakistan, it reached 450 per 100,000 [4].

In recent years, healthcare professionals have witnessed the rapid rise of multi-drug resistant (MDR) and extensive drug-resistant (XDR) *Salmonella enterica* serotype Typhi [5][6]. Typhoid fever that is resistant to traditional first-line antibiotics including ampicillin, chloramphenicol, and trimethoprim-sulfamethoxazole is regarded as MDR typhoid [2]. XDR typhoid has resistance to fluoroquinolones and third-generation cephalosporins [7]. Several factors have played a role in the emergence of antibiotic-resistant typhoid in Pakistan, such as restricted access to clean water and sanitation facilities, as well as insufficient awareness about the disease [7]. Furthermore, the absence of vaccination and unrestricted availability of various antibiotics have also been contributing factors. In addition, the practice of initiating antibiotic treatment empirically before confirming the diagnosis through blood culture has further exacerbated the issue of increased resistance [8,9].

Due to the emergence of XDR typhoid fever, these antibiotics are also facing resistance. Therefore, it is crucial to study the resistance patterns of *Salmonella enterica* serotype Typhi in this region. The cross-sectional study will focus on antibiotic resistance of *Salmonella* in Pakistan using statistical analysis in response to various antibiotics while also comparing comorbidities and resistance to *Salmonella enterica* serotype Typhi.

## Materials and methods

Study design

The database observational study was conducted on blood cultures submitted to the clinical microbiology laboratory of Khyber Teaching Hospital (KTH) from September 2021 to August 2022.

Sample size

The sample size was calculated using the World Health Organization (WHO) sample size calculator (https://cdn.who.int/media/docs/default-source/ncds/ncd-surveillance/steps/sample-size-calculator.xls). The recommended sample size was 385, but to ensure robust statistical power and representativeness, data from a much larger cohort comprising 17,632 blood cultures were included in the analysis. 

Data collection and management

The hospital database was reviewed for the results of blood cultures and sensitivity reports of patients suspected of enteric fever. Both inpatient and outpatient data were reviewed. Only data from patients whose blood culture was positive for *Salmonella* Typhi species was included in the study. The hospital laboratory used agglutination tests for differentiating serotypes of *Salmonella enterica*. Furthermore, the patients' demographic information, including age, gender, history of previous *Salmonella* Typhi infection, antibiotics used in the last three months, and other comorbidities, were recruited from the database. The study excluded data from patients with bacteremia caused by *Salmonella* Paratyphi due to the limited number of cases. In addition, data from blood culture reports done for conditions other than enteric fever were excluded from the study. The hospital laboratory used the BACTEC system for growing *Salmonella* Typhi species, and antibiotic susceptibility testing was performed using the Kirby-Bauer disc diffusion technique on Muller-Hinton agar.

Data analysis

The data received from the laboratory were recorded in Microsoft Excel (Microsoft Corporation, USA) and were organized and stored in a spreadsheet format for further analysis. Data were transferred from Microsoft Excel sheets to IBM SPSS Statistics for Windows, version 25.0 (released 2017, IBM Corp., Armonk, NY). Frequency analysis was conducted on variables, such as gender, age, previous history of *Salmonella* infection, antibiotics used in the last three months, and comorbidities. Resistance and sensitivity rate was calculated and cross-tabulation was utilized to explore potential associations between different variables, with statistical significance assessed using both Pearson's chi-square test and Fisher's exact test. A significance level of p < 0.05 was deemed indicative of statistically significant findings.

Ethical consideration

Ethical approval was taken from Khyber Medical College Peshawar Institutional Research and Ethical Review Board (IREB) with reference no. 688/DME/KMC, dated September 21, 2022.

## Results

A total of 504 blood culture reports were reviewed in the study. Thirty-six percent (n = 182) were female, 64% (n = 321) were males, and 0.2% (n = 1) were neuter gender. The mean age of the patients was 12.92 years, with a standard deviation of 11.85. Eight percent (n = 40) of the patients had comorbidities, while 92% (n = 464) did not. Ninety-two percent (n = 463) of the patients did not use antibiotics for *Salmonella* infection in the last three months, while 8% (n = 41) did. Sixty-nine percent (n = 349) of the patients did not use antibiotics for any indication, while 31% (n = 155) used antibiotics for reasons other than *Salmonella*.

**Figure 1:**
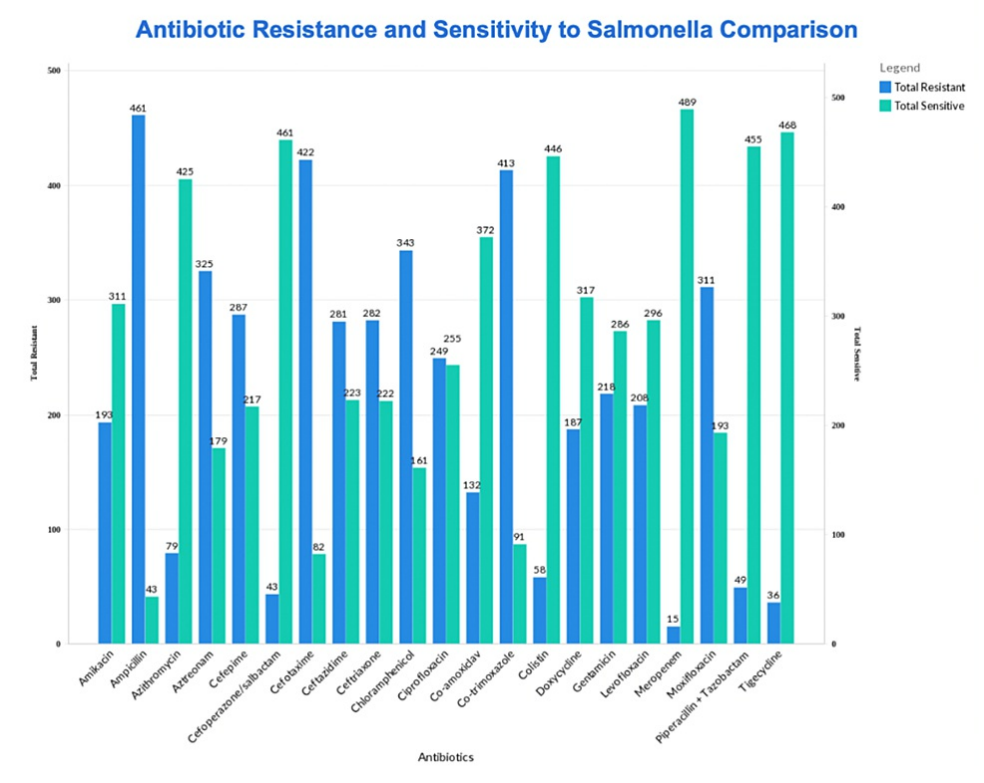
Patterns of antibiotic resistance and sensitivity to Salmonella Typhi

In terms of resistance, ampicillin had the highest resistance rate (91.47%), followed by cefotaxime (83.7%) and co-trimoxazole (81.9%). In terms of sensitivity, meropenem had the highest sensitivity rate of 96.4%, followed by tigecycline (92.8%) and cefoperazone/sulbactam (91.4%). The detail of sensitivity and resistance patterns to various antibiotics is shown in Figure 1.

**Figure 2:**
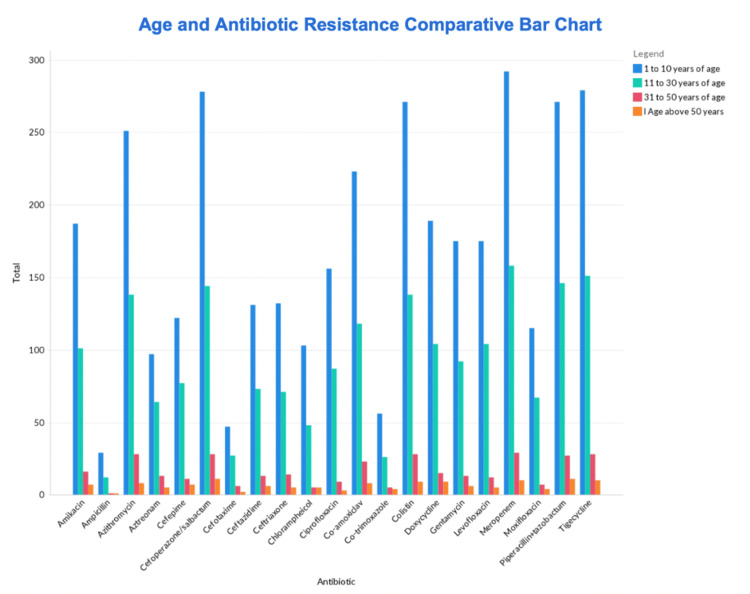
Comparison of antibiotic sensitivity to Salmonella Typhi based on the age of the patient

Among the age groups analyzed, ampicillin demonstrated the highest sensitivity in the 1-10 age group (67.4%), followed by aztreonam in the 11-30 age group (35.8%). In the 31-50 age group, cefotaxime and aztreonam exhibited sensitivity rates of 7.3% each, while co-trimoxazole had the highest sensitivity in the 50+ age group (4.4%). Although these variations were observed, statistical analysis indicated that the differences were not statistically significant (p > 0.05). The summary of age-wise analysis is shown in Figure 2.

Among the antibiotics analyzed, several exhibited significant p-values, indicating a statistically significant relationship with the outcome variable. These antibiotics include ceftazidime (p = 0.038*), cefepime (p = 0.005*), aztreonam (p = 0.006*), and cefotaxime (p = 0.017*). Cefotaxime and co-trimoxazole exhibited p-values less than 0.05, suggesting a substantial difference in the sensitivity of *Salmonella* in patients with comorbid conditions. This finding highlights the importance of considering the presence of comorbidities when selecting the appropriate antibiotic therapy for *Salmonella* infections. Moreover, Fisher's exact test revealed that meropenem also had a p-value less than 0.05. This result further emphasizes the difference in *Salmonella *sensitivity among patients with comorbidities, specifically when treated with meropenem.

Further analysis conducted using the chi-square test revealed significant differences in antibiotic sensitivities among patients who had previously used antibiotics for *Salmonella* infections in the last three months. Specifically, ceftriaxone, moxifloxacin, gentamycin, amikacin, and levofloxacin showed significant differences. In addition, Fischer's exact test indicated significant differences in sensitivities to piperacillin-tazobactam, meropenem, colistin, and tigecycline. These findings provide strong evidence of a notable variance in antibiotic responses among patients with a recent history of *Salmonella *infections.

## Discussion

Typhoid fever is a systemic febrile disease that requires immediate antibiotic treatment. Drug-resistant typhoid strains have imposed a great challenge on healthcare systems, both in Pakistan and the rest of the world. Many studies point to the lack of access to healthcare services along with the increased supply of over-the-counter (OTC) drugs as one of the main contributors to antibiotic resistance in lower-middle-income countries [5]. In 2016, extensively drug-resistant *Salmonella* Typhi was isolated in Pakistan and has been responsible for multiple outbreaks in Pakistan and many travel-related cases all over the world [6]. In this study, we have observed the rate of resistance and sensitivity of various antibiotics in the case of *Salmonella *Typhi.

It was observed in our study that the demographics were mostly males and the mean age was 12 years old. This demographic was consistent with many similar studies and is possible because, in Pakistani society, males have more outdoor exposure and are more likely to consume street foods as compared to females [10]. Among the treatment regimens for *Salmonella*, it was noted that historically recommended regimens, such as ampicillin, chloramphenicol, and co-trimoxazole, showed the highest resistance (91.5%, 68.1%, and 81.9%) with decreased sensitivity (8.5%, 31.9%, and 18.1%, respectively). This result was similar to that of another study conducted in Pakistan by Aslam et al. [11], which indicated similar sensitivity rates as our study and also indicated a decrease in sensitivity among older antibiotics. Due to resistance in the older drugs, ciprofloxacin (fluoroquinolone) became the current first-line antibiotic [12]. Over the years, the overuse of this antibiotic caused an increase in the development of antimicrobial resistance toward fluoroquinolones. This was also reflected in our study, where ciprofloxacin showed a 49.4% resistance rate and 50.6% sensitivity against the *Salmonella* Typhi species. The developing resistance and decrease in sensitivity have caused the shift toward the use of third-generation cephalosporins and azithromycin as the new drugs of choice [9].

Among the cephalosporins, ceftriaxone, ceftazidime, and cefepime showed similar rates of efficacy with almost equal resistance and sensitivity in our study. The only exception was cefotaxime, which showed a much higher resistance (83.73%) and lower sensitivity (16.27%). These findings suggest the emergence of resistance and decreased sensitivity to current first-line antibiotics used against *Salmonella*. Our study highlighted promising results for meropenem, tigecycline, cefoperazone/sulbactam, piperacillin + tazobactam, colistin, and azithromycin. However, it is worth noting that some studies have indicated growing concerns about azithromycin resistance and its relation to treatment failure [13].

*Salmonella* Typhi isolates are defined as MDR if they are resistant to chloramphenicol, ampicillin, and trimethoprim-sulfamethoxazole and as XDR if they are MDR, nonsusceptible to fluoroquinolones, and resistant to third-generation cephalosporins. The outbreak of XDR Salmonella Typhi resistant to both cephalosporins and fluoroquinolones, which was reported in Pakistan during 2016-2018, was found to be only susceptible to azithromycin and carbapenems [9]. According to our findings, meropenem was seen to be the most effective, with 3% resistance and 97% sensitivity, which is consistent with the above-mentioned study. This could indicate that this XDR Salmonella Typhi strain is still the predominant one in Pakistan.

Furthermore, a similar study conducted in Bangladesh revealed that resistance is more prevalent among adults (between 30 and 40 years) and children (between 0 and 10 years) [14]. Our findings indicate that there was no statistically significant difference in terms of resistance and sensitivity observed among the age groups investigated in our research. In addition, our study population did not demonstrate any statistically significant disparity between males and females. One of the major findings in our study shows that the patients who had prior antibiotic use against *Salmonella *infections showed reduced sensitivity rates compared to patients without. The results of a prospective study conducted in Bangladesh, Nepal, and Pakistan reported that one out of every three participants had evidence of antibiotic activity in their urine assay upon presenting at the tertiary care study site. It was interesting to note that patients who had taken antibiotics prior to presentation were more likely to have a positive culture of *Salmonella* than those who did not [15]. Similarly, our study also revealed that patients with comorbidities displayed lower sensitivity rates to antibiotics than those without comorbidities. This finding is supported by evidence in the literature, which suggests that chronic conditions like diabetes and cardiovascular disease put patients at a higher risk for MDR infections [16].

Some limitations of our study include the absence of a control group, our retrospective data collection method, and the use of a single-center study design. The lack of a control group could hamper the ability to establish causality or determine treatment effectiveness. Relying on retrospective data collection and a single-center approach introduces potential biases and restricts the generalizability of findings. Future research should address these limitations by including control groups, using prospective data collection, and involving multiple centers for more reliable and comprehensive results.

## Conclusions

Multiple studies have revealed that the management of MDR and XDR strains of *Salmonella* Typhi in Pakistan has become an immense challenge for the healthcare system. The efficacy of numerous drugs in treating this disease has significantly declined. Currently, carbapenems have emerged as the last line of defense in resistant cases. Consequently, it is imperative to implement various strategies to combat this escalating issue and hinder the emergence of further resistant strains. Promoting rapid diagnostics, colonization prevention, uniformity in antibiotic usage, consideration of combination therapy with a high sensitivity rate, and shorter duration of antibiotic courses are among the key strategies that should be emphasized in Pakistan. By complementing these measures with a stricter regulation of over-the-counter antibiotics, which are easily accessible to the general population, we can effectively reduce antibiotic resistance.
